# Role of Cytokinins for Interactions of Plants With Microbial Pathogens and Pest Insects

**DOI:** 10.3389/fpls.2019.01777

**Published:** 2020-02-19

**Authors:** Saqib Saleem Akhtar, Mengistu F. Mekureyaw, Chandana Pandey, Thomas Roitsch

**Affiliations:** ^1^ Department of Plant and Environmental Sciences, University of Copenhagen, Copenhagen, Denmark; ^2^ Department of Adaptive Biotechnologies, Global Change Research Institute, CAS, Brno, Czechia

**Keywords:** host–pathogen interaction, beneficial microbe, plant growth promoting rhizobacteria, microalgae, insect, phytohormones

## Abstract

It has been recognized that cytokinins are plant hormones that influence not only numerous aspects of plant growth, development and physiology, including cell division, chloroplast differentiation and delay of senescence but the interaction with other organisms, including pathogens. Cytokinins are not only produced by plants but are also by other prokaryotic and eukaryotic organism such as bacteria, fungi, microalgae and insects. Notably, cytokinins are produced both by pathogenic and also beneficial microbes and are known to induce resistance in plants against pathogen infections. In this review the contrasting role of cytokinin for the defence and susceptibility of plants against bacterial and fungal pathogen and pest insects is assessed. We also discuss the cross talk of cytokinins with other phytohormones and the underlying mechanism involved in enhancing plant immunity against pathogen infections and explore possible practical applications in crop plant production.

## Introduction

Plant hormones (Phytohormones) are naturally occurring small organic molecules that affect numerous aspects of growth and differentiation in plants and are involved in alleviating different biotic and abiotic stresses (Davies, 2010). So far, nine categories of phytohormones have been identified. These include auxins, cytokinins (CK), gibberellins (GA), abscisic acid, (ABA), ethylene (ET), brassinosteroids (BR), salicylates (SA), jasmonates (JA), and strigolactones (SL) (Su et al., 2017). These hormones are not only produced by plants but are also produced by beneficial and pathogenic microorganism (e.g. bacteria, fungi, insects, microalgae etc.) that may modulate plant growth, physiology and immunity. Among phytohormones, ET, SA, and JA are particularly known for regulating defence response in plants against pathogens and are called as the immunity hormones. However, recently the role of cytokinin in inducing immunity in plant upon plant-pathogenic interaction has also been recognised (Grosskinsky et al., 2011; Grosskinsky et al., 2016; Naseem et al., 2014; Spallek et al., 2017; Dowd et al., 2017; Siddique et al., 2015; Shanks et al., 2016).

Cytokinins (CKs) influence various traits of plant growth, development and physiology such as seed germination, apical dominance, flower and fruit development, leaf senescence and plant-pathogen-interactions etc. CKs are isoprenoid substituted adenines molecule. Isopentenyltransferases (IPTs) is the first enzyme involved in catalyzing isoprenoid to other various types of CKs including cis-zeatin (cZ), N6-(Δ2-isopentenyl)-adenine (iP), trans-zeatin (tZ), and dihydrozeatin (DZ) (10). In plants, tZ occur in the most abundant form. CKs are further metabolized and inactivated through conjugation to sugars or through degradation by CK oxidases (CKXs).

The type and activity of CK molecules differ remarkably between different plant species and tissues, at different developmental stages and under various environmental conditions. CKs are not only produced by plants but are also produced by plant associated microorganism, microalgae and insects.

An increasing experimental evidence support the role of CKs in enhancing plant resistance against plant pathogen such as bacteria, fungi and pest insects (**Table 1**). A role of CKs for interaction with insects is known for decades and the findings of CK mediated resistance against microbial pathogens in *Arabidopsis* (Choi et al., 2010) and tobacco (Grosskinsky et al., 2011) have been extended also to other species (Naseem et al., 2014; Siddique et al., 2015; Shanks et al., 2016; Dowd et al., 2017; Spallek et al., 2017).

**Table 1:** Summary of research findings indicating the effect of cytokinin on plant pathogen interactions.

Pathogen type	Pathogen	Host plant	Source of CK	Effects	Reference
Bacteria	*Pseudomonas syringae*	*Arabidopsis thaliana*	*Pseudomonas fluorescens*	Cytokinin producing *P. fluorescens* G20-18 can be used as a novel biocontrol agianst *P. syringae* infection	Grosskinsky et al. (2016)
	*P. syringae pv.*	*A. thaliana*	Zeatin (chemical source)	Exogeneous application of 1 μm of trans-zeatin induce resistance in arabidopsis against *P. syringae* pv.	Choi et al. (2010)
	*P. syringae pv. Tabaci (PsT)*	*Nicotiana tobacum*	Exogenous application of kinetin (1–18 µM) and endogenous increase (i.e. upregulation of IPT)	Application of 10 µM of Kinetin to detached leaves of tobacco for 24h enhanced resistance against Pst T infection by up to 95%.	Grosskinsky et al. (2011)
Fungi	*Erysiphe graminis* f. sp. tritici	*Triticum aestivum*	Exogenous trans-zeatin	Cytokinin-induced immunity and cytokinin-induced susceptibility	Babosha (2009)
	*Magnaporthe oryzae*	*Oryza sativa* subsp. japonica	Exogenous kinetin or isopentenyladenine (1–100 lM) plus SA analogue	Cytokinin-induced immunity	Akagi et al. (2014), Jiang et al. (2013)
Insect pest	Gypsy moth (*Lymantria dispar*)	*Populus*	Exogenous application of Benzylaminopurine (BAP) 100 µM., (Totally 25 ml per Plant)	Daunt insect feeding, delay larval development or reduce weight gain by insect larvae, wound-inducible accumulation of JA and LNA	Dervinis et al. (2010)
	Specialist herbivore *Manduca sexta*	*N. attenuata*	Endogenous	Stimulate cytokinin signaling in wild tobacco based on elevated abundance of transcripts for *cig2* (a cytokinin-induced gene)	Hui et al. (2003)

A potential dual role of fungal and microalgae produced CKs in modulating host immunity and optimizing nutrient supply finds experimental support (Chanclud et al., 2016; Uthirapandi et al., 2018). Likewise, bacterial produced CKs induce resistance in *Arabidopsis* against bacterial pathogens (Grosskinsky et al., 2016). CK can also prime plant responses to insect herbivory attack by stimulating wound-inducible gene expression and by inducing accumulation of insecticidal compounds (Dervinis et al., 2010; Giron et al., 2013).

CK altered transcript levels in the CK regulatory pathways revealed genes associated with light-responses, cold-inducible COR genes, syncytial endosperm developmental genes CKX1, CKX2, IPT4, and IPT8 as well as CK receptors AHK2 and AHK3 (Liu et al., 2013; (Song et al., 2015). Thus, epigenetic controls might integrate inputs from developmental and metabolic control to stress tolerance. Furthermore, genetic pathway and epigenetic regulations coordinates the action of genetic and epigenetic factors regulating adaptation towards CK regulation and allow plants to adapt favourable resources available from their environment. In this context, modern genome editing tools could be employed to target and manipulate CK levels in both plant and beneficial microbe to fight against pathogen with the concurrent aim of maintaining quality. Thus, further research is needed to investigate the expression of the IPT and CKX gene family members as well as of genes involved in source-sink relationships during leaf, flower, and silique development during coordinated action of CK production through beneficial microbe and plant against pathogen.

Increased seed yield in variety of plants has been observed with ectopic expression of IPT gene [see review by (Guo and Gan, 2014)] and endogenous CKs are routinely detected in developing fruits and seeds. However, speculation on the role of CKs in chromatin remodelling to understand the processes responsible for establishing and maintaining gene expression patterns in plant immunity is quite an interesting topic now a days.

In addition to plants, beneficial microbes such as plant growth promoting rhizobacteria (PGPR) and microalgae, CKs are also produced by plant pathogens like fungi (Chanclud et al., 2016), nematodes (Siddique et al., 2015; Shanks et al., 2016; Dowd et al., 2017), phytoplasma (Dermastia, 2019) and parasitic plants (Spallek et al., 2017). The CKs from these plant pathogenic organisms have contrasting effect on plant growth and may involve hijacking plant defence and enhances disease virulence (Spallek et al., 2018). However, the focus of this review is only on the role of CKs for interaction with pathogenic bacteria and fungi as well as pest insects. Within these interactions we discuss the cross talk of CKs with other hormones in inducing plant resistance against pathogens and explore possible practical applications in plant protection.

### Role of Cytokinin for Interaction of Plants With Bacterial Pathogens

Higher level of CK in plant increased resistance to pathogens whereas opposite is true for plant susceptibility to diseases (Choi et al., 2010; Grosskinsky et al., 2011; Naseem et al., 2014; Grosskinsky et al., 2016; Albrecht and Argueso, 2017). Sufficient evidences are available in literature indicating the role of exogenously applied CK on altering the level of host resistance to pathogen (Grosskinsky et al., 2011; Naseem et al., 2014; Grosskinsky et al., 2016; Cortleven et al., 2019). For instance, Choi et al. (2010), treated *Arabidopsis* with 1 μm of trans-zeatin (naturally occurring CK), for which the CK receptors have very high affinity, in response to the bacterial pathogen *Pseudomonas syringae* pv. tomato (Pst) DC3000. Transient overexpression of CK producing IPT genes in tobacco (Grosskinsky et al., 2016) plant resulted in increased resistance against *P. syringae* whereas, overexpression of CK oxidase encoding gene leads to increase plant susceptibility.

Since the discovery of CK in plant, it has been assumed that these phytohormones are produced by plant. However, Holland (1997) hypothesized that CK are synthesized by endophytic methylotrophic pink-pigmented facultative methylotrophic (PPFM) bacteria in plants rather than plant themselves. If this was the case, CKs could not be considered as plant hormones (Romanov, 2011). However, presence of sufficient experimental approaches and material provided enough proofs and evidences to disprove this hypothesis. For example, CK moieties in certain tRNA species of a different organisms (including plants), production of CKs by different microorganisms and the ability of bacteria to transform cells of the host to synthesize CKs, as well as the production of CKs by untransformed plants are the key findings to disprove Holland's hypothesis (Kamínek, 2015). On the other hand it is also very clear that CK is produced by plants, insects, microorganisms, nematodes, and parasitic plants. Recent studies revealed that endosymbiotic methylotrophic bacteria contribute to abiotic stress resistance *via* increasing plant CK levels (Jorge et al., 2019; Kumar et al., 2019).

A spoon of soil can hold up to billions of bacteria. These root associated prokaryotes may have either beneficial, detrimental or neutral effect on plants. Most of the plant pathogenic bacteria belong to genera: *Agrobacterium, Erwinia, Pectobacterium, Pseudomonas, Pantoea, Burkholderia, Ralstonia, Xanthomonas, Spiroplasma, Clavibacter, and Phytoplasma*. These bacteria may be recognised by causing different plants symptoms e.g include gall, wilt, leaf spot, overgrowth, soft rots etc upon their infection. On the other hand, most common plant beneficial bacteria belong to genera: *Bacillius, Pseudomonas, Bradyrhizobium, Agrobacterium, Enterobacter, and Burkholderia* etc. (Ryu et al., 2004; Maksimov et al., 2011; Wang et al., 2012; Gururani et al., 2013; Akhtar et al., 2015; Belimov et al., 2015; Kanwal et al., 2017; Nadeem et al., 2017; Zhou et al., 2017; Cordero et al., 2018; Ishizawa et al., 2019). These PGPR promote plant growth through either direct or indirect mechanism of action (Nadeem et al., 2014). Direct growth promotion take place in different ways like by providing beneficial compound to the host plant synthesized by bacterium and/or by facilitating nutrient uptake from soil environment (Nadeem et al., 2014; Cordero et al., 2018). While indirect growth may occur when microbes prevent or reduce the virulence of pathogenic microbes by producing antimicrobial products or by increasing resistance in plants against pathogens (Glick and Bashan, 1997; Maksimov et al., 2011). In addition, PGPR has been well known for their production of phytohormones e.g. auxin, ET and CK (Castillo et al., 2015; Maheshwari et al., 2015).

PGPR can also synthesize CK (Arkhipova et al., 2007; Liu et al., 2013). PGPB can enhance the level of CK concentration of soil solution and of plants growing there. Hence, plants inoculated with CK producing bacteria (Liu et al., 2013) promote growth in similar fashion as applied exogenously. For instance, Liu et al. (2013) observed increase in root and shoot dry biomass of *Platycladus orientalis* by CK producing *Bacillus subtilis.* Similar findings were reported by Arkhipova et al. (2005) where bacterial inoculated lettuce plants increased ten time more zeatin and riboside content in roots than in control shortly after two days of inoculation.

CK producing PGPR can not only be use as bio-stimulant for plant growth but can also be used for biocontrol for different pathogen. Priming of *Arabidopsis* with CK producing *Pseudomonas fluorescens* G20-18 efficiently controls the impact of the hemibiotrophic bacterial pathogen *P. syringae* while CK deficient loss of function mutant of G20-18 exhibited impaired biocontrol against pathogenic bacteria (Grosskinsky et al., 2016). Relatively higher level of CK in phytoplasma-infected plant roots, stems and flowers suggests its role against this class of bacterial pathogen. Also, different plants infected with phytoplasma shows different characteristics like up-regulation of the CK biosynthetic gene encoding isopentenyl transferase and down-regulation of CK biodegradation gene encoding CK oxidase (Dermastia, 2019).

*Agrobacterium tumefaciens* is a soil bacterium that has the ability to transfer foreign genes in host plant cells. *It* introduces T-DNA into genome of various plant species and causes crown gall disease (Arkhipova et al., 2007) mediated by the expression of CK and auxin in the plant. The Ti plasmids of nopaline-type *Agrobacterium* strains carry a tzs gene which involves in production and secretion of CKs also by the bacterium (Regier and Morris, 1982). Tzs stimulate transformation by both nopaline-type *A. tumefaciens* strains (Hwang et al., 2010) and, when transferred to strain 1855, *A. rhizogenes* strains. *A. tumefaciens* strains harboring nopaline-type Ti plasmids secrete trans-zeatin or trans-zeatin (Hwang et al., 2010). In addition, all *Agrobacterium* strains produces CKs from derivatives of isopentenylated transfer RNA (tRNA) (Hwang et al., 2012; Sardesai et al., 2013). According to Sardesai et al. (2013), CK produced by *Agrobacterium* played a critical role in promoting transformation in *Arabidopsis* by repressing plant Myb transcription factor. The Gram positive bacterium *Rhodococcus fascians* causes leafy galls symptoms in plants which resemble to the symptoms caused by exogeneous applied CK to plants. Moreover, CK pathways downstream of AHK3 and AHK4 are similarly important for leafy gall formation (Jameson et al., 2019). However, CK-induced susceptibility is interconnected to an early, AHK3 and AHK4-dependent, transcriptional reprogramming that renders host cells more receptive to *A. tumefaciens* (Sardesai et al., 2013).

### Role of Cytokinin for Interaction of Plants With Fungal Pathogen

In recent years, fungal disease owing to growth abnormalities, physiological and morphological alterations, and varying distribution of carbon source is one of the major environmental biotic factors negatively affecting plant health (Mishra et al., 2018). Biotrophic fungal plant pathogens derive their nutrition from living host cells, a feature which distinguishes them from the necrotrophic fungi that obtain their nutrients from host tissues which they have killed. Both of these fungi group are able to ultimately kill host cells at early stages and causes alteration/change in plant cellular and physiological responses, such as enzyme activity changes, stomata closure, and alteration in gene expression (Pusztahelyi et al., 2016). During fungal attacks, plants trigger a hypersensitive activity by regulation of CK biosynthetic gene IPT in the fas operon and kills cells near the infection site and prevent them to spread by creating pathogen nutrient deprives environment (Novák et al., 2013). Thus, CK producing biotrophs and hemibiotrophs regulate the host cell processes that are prerequisite for pathogenesis such as cycle and nutrient allocation by manipulating CK signaling. In addition to plants-fungus interaction, CK alterations have been reported for galls and green island formation, abnormalities in plant growth (Walters et al., 2008), and modulation of primary carbon metabolism. Tumor-inducing fungal pathogens are able to produce CKs similar to distinct fungi *Ustilago maydis* (Morrison et al., 2015), *Fusarium pseudograminearum* (Sørensen et al., 2018) and *Claviceps purpurea* (Hinsch et al., 2015) etc. Choi et al., 2010 investigated the CKs regulation on a virulent necrotrophic fungus, *A. brassicicola* KACC40036 and observed that overexpression of transcriptional activators IPT3 or ARR2 in CK signaling, led to enhanced *A. brassicicola* resistance. Thus, CK may activate several defensce network by priming affect in plants and thus elevating resistance to different fungal pathogens. Moreover, findings of Jiang et al. (2013) regarding CK accumulation determines the activation of defence gene PR on pathogen infection in rice plants by synergistic interaction of CK with SA. in *Arabidopsis* similar activity of CK have been observed, where CK modulated the SA signaling pathway and increased the resistant activity of *P. syringae* pv. tomato DC3000 and *H. arabidopsis* Noco2 (Choi et al., 2010; Argueso et al., 2012). In *F. mangiferae* infected maize plant, changes in CK levels are related to vegetative malformations and inhibition in growth (Vrabka et al., 2018).

The significant role of CK and its interaction in plant and fungus was elucidated by the study of biotrophic fungus ergot *Claviceps purpurea*. It synthesizes a variety of CKs which includes cZ‐type CKs, the predominant form of CKs in rye, the main host of *C. purpurea* (Hinsch et al., 2015). While mutation in CK synthesis genes of *C. purpurea* strains, specifically strains with abolished cZ-type CKs, and expressing CKX gene showed less virulence activity (Hinsch et al., 2015; Kind et al., 2018). However, some other reports revealed that CKs enhance the plants resistance to pathogens that do not secrete CKs by modulating defence signaling. Liu et al. (1986) reported inhibitory effects of exogenous supply of kinetin on fungus development. In addition, pathogen infection is responsible for the accumulation of various class of CKs in resistance and susceptible varieties. Plant infected with *P. teres* and *D. maydis* results in maximum 6- Y,Y-dimethylall ylaminopurine in susceptible barley and maize, however, in infected resistant hosts zeatin/zeatin riboside were maximum among the different class of CKs (Angra-Sharma and Sharma, 2000). CK producing bacteria could be cultivated and explored as a biofertilizer against fungal pathogen (Mishra et al., 2018). The CKs detected in *Magnaporthe oryzae* infected rice leaves act as a defence signal to mobilize nutrients, to increase levels of photosynthesis in host leaves or to activate SA-mediated defence responses (Jiang et al., 2013). *M. oryzae* strains deleted in encoding gene CK Synthesis 1 (CKS1) of tRNA-IPT, produce less lesions than wild-type strain without exhibiting distinct growth and development deficits in phenotypic appearance of rice plants (Chanclud et al., 2016). However, exogenously CKs application restored the reduced virulence of cks1 mutants.

Dictated by evolution, plants adapt to the various environmental stresses, so that assisting plant acclimation and survival. For this a tight and efficient coordination of the varied responses and further adjustments in adaptive way, including alteration of root-shoot ratio (R/S), phytohormone equilibrium, regulation of photosynthesis, and photoassimilate translocation. CKs produce by plants, well known for cell division, leaf longevity and nutrient mobilization (Choi and Hwang, 2007) are considered to attribute plant immunity *via* SA signaling (Choi et al., 2010). Different investigations support that CKs have a wide range of functions in plant–pathogen interactions. The classic phytohormone family of CKs with growth-stimulating activity consist of important regulators of many of these fungal–bacterial pathogens induced physiological and developmental plant processes (Grosskinsky et al., 2016; Spallek et al., 2018).

### Role of Cytokinin for Interaction of Plants With Pest Insects

Resource allocation in plants for growth and defence needs to be regulated efficiently (Pieterse et al., 2014). Recent findings in plant related researches have indicated that phytohormone signaling networks play role in interconnecting growth, development and defence, making plants to choose when to invest their resource; under favourable condition in growth and development or in defence when they are exposed to biotic or abiotic stresses (Pieterse et al., 2014; Albrecht and Argueso, 2017). Metabolic reprogramming and source to sink ratio of resources could be responsible for growth and development limitation during defence priming (Albrecht and Argueso, 2017), several lines of experimental evidence showed that this kind of scenario may not indicate the full picture of growth–defence trade-offs (Bennett et al., 2012; Giron et al., 2013; Albrecht and Argueso, 2017).

Beneficial microbes are recognized to induce systemic resistance against herbivorous insects and prevention to different environmental stresses (Giron and Glevarec, 2014; Pieterse et al., 2014). Living factors like insect and pathogens and environmental factors like drought and salinity are known to influence microbial synergy by changing the plant physiology and exudating root sap (Bennett et al., 2012; Giron et al., 2013; Pangesti et al., 2013). It has been known long time ago that specific defence gene activation was regulated by different plant hormones; like JA, SA and ET (Moreno et al., 2014). Moreover, it has been revealed that important defence regulations are carried out by other phytohormones like; ABA, GA, auxins and CK (Giron et al., 2013).

CK can start plant reaction to wound and insect attack by initiating wound-inducible gene expression and by inducing increase of compounds against insect (Dervinis et al., 2010; Giron et al., 2013). This kind of wound response may indicate physiological and metabolic signals of CK could interfere on anti-herbivore defence in foliage (Dervinis et al., 2010). CK signaling is also related to macro and micro-nutrient and nitrogen availability, which can put critical influence on plant and insect growth and development (Dervinis et al., 2010). Higher concentration of CK can provide to tissue repair by inducing cell division (Giron et al., 2013). CK mediated insect resistance has been reported with different phenomena; like daunt insect herbivory, slow larval growth, or minimum weight increase by insect larvae in tobacco hornworm *Manduca sexta*, the gypsy moth *Lymantria dispar* or the green peach aphid *Myzus persicae* (Dervinis et al., 2010; Giron et al., 2013).

CK is the reason for green island establishment in insect attack based on much amount in infected parts (Engelbrecht et al., 1969). It was first shown in mining microlepidopterans, then the green islands around the galls of phytophagous hymenopterans, psyllid homopterans, cecidomiids, and tephritid dipterans was due to increased level of CK (Engelbrecht et al., 1969; Lisabeth, 1971; Elzen, 1983). Endophytic insects mold the host plant's physiology, repetitively changing source/sink proportion by moving CK to create this phenomenon which increases the nutritional value of infested tissues. It was clearly shown when free living insect *Tupiocoris notatus* was feeding on *Nicotiana attenuata* by stable nutrient levels, increased CK levels and alterations in CK-related transcript levels in attacked leaves (Brütting et al., 2018).

Higher amount of different CKs (zeatin, isopentenyl adenine, and isopentenyl adenosine) was shown on infected tissues of *Malus domestica/Phyllonorycter blancardella* leaf-mining system. This is clear indication of the “stay-green” phenomena of attacked areas, whereas the rest the same leaf are changing to yellow, in addition the net accumulation of minerals in infected tissues in a very specific design answering the energetic need of the growing larvae (Giron et al., 2007; Giron et al., 2016). Increase in the concentration of isopentenyl adenine was reported in galls inferred by larvae of *E. solidaginis* (Jameson, 2000; Mapes and Davies, 2001; Sakakibara, 2006).

It is known that production of CK by gall-producing bacteria and green island forming fungi, but localized delay of senescence and formation of stay green phenomena on insect-attacked leaves could be due to production of CK by insects (Walters et al., 2008; Kaiser et al., 2010). Finding high level of CK in insect excretion, gastrointestinal span and minor salivary glands of leaf-feeding larvae of Caterpillar suggests their potential for CK production (Engelbrecht et al., 1969). Insect exudates or glands related with oviposition and induction of galls, shows again that insect larvae may use as source of CKs (Elzen, 1983; Giron et al., 2016). When in fact the formation of stay-green phenomena by insect herbivores exploiting CK levels is known for decades, the reaction of CK pathway to chewing insect herbivory has been known only more recently. Different research output are now indicating CK are main part of wounding and herbivore-associated molecular patterns (HAMPs) induced responses in many plant species. (Schäfer et al., 2015).

The detailed analyses of movement and source of CKs participated in plant feeding by leaf-mining insects has revealed that the insect symbiotic bacteria *Wolbachia* is inherently involved (Zhang et al., 2017). This complicated plant-insect-microbe interaction is essential and the modulation of plant CK levels was shown to be impaired in the *Wolbachia*-free leaf feeding moth (Zhang et al., 2018).

### Cross Talk Between Cytokinins and Other Hormones Within Plant Immune Responses

Nowadays there are many research outputs on how actions of different phytohormones are making a signaling interaction to regulate different signaling processes and metabolic systems, which are important in plant development and responses for biotic and abiotic stress (Jiang et al., 2013; Munné-Bosch and Müller, 2013; Joseph et al., 2018). Previous researches greatly increased our understanding of how hormones influence plant growth and development and also their response to different stresses, it is now very clear that physiological processes are controlled in a complex interaction by cross-talk of several hormones (Munné-Bosch and Müller, 2013).

To mention some research outputs on how plant hormones are making signaling network, cross-talk of auxin and GA in plant growth regulation; interaction of CKs, auxin, ABA and SL in apical dominance; auxin and BR in cell expansion; ET and CKs in root inhibition and hypocotyl elongation; SA, JA and auxin interaction in plant pathogen response and cross-talk between ET, ABA and GAs in plant responses to different stresses (Munné-Bosch and Müller, 2013; O'Brien and Benková, 2013).

Plant growth, development and adaptations for different biotic and abiotic stresses are the result of intricate network of many synergistic and antagonistic cooperation between different hormones (O'Brien and Benková, 2013). Crosstalk between different phytohormones is crucial during interaction of plants with different stresses (Jiang et al., 2013). This complicated interaction of hormone signaling pathways makes plants to induce the required and effective defence reactions against pathogens and also to balance defence and plant growth (Berens et al., 2017). It has been long time since CK is known for its importance in plant growth and development and the implication in plant defence response have been realized more recently (Naseem et al., 2013). Dealing in this aspect will reveal key biological conclusion having to do with the trade-off between growth and defence (Naseem and Dandekar, 2012; Naseem et al., 2013).

It has been known that central backbone of plant defence response is the result of interaction between JA, SA and ET (Naseem and Dandekar, 2012; Naseem et al., 2013). CK advances SA mediated of plant defence but does not help JA-dependant responses during *Arabidopsis* infection by *P. syringae* pv tomato DC3000. This indicates positive interaction between CKs and SA and negative interaction between JA and CKs in adjusting immune response of *Arabidopsis* during the infection (Naseem et al., 2013). Furthermore, CK and SA play key role in activating defence gene expression by rice against infection with blast fungus (*Magnaporthe oryzae*) (Jiang et al., 2013). It is also reported about co-regulation of CK level with SA level when tobacco plants (*N. tabacum*) are infected with *tobacco mosaic virus* (TMV), *cucumber mosaic virus*, potato virus X, and potato virus Y (O'Brien and Benková, 2013). Generally high level of CK can modulate SA signaling and increase protection against viruses and bacteria through higher expression of SA-related defence genes (Jameson, 2000).

The SA-dependence of CK-induced immunity can support inducing activity of CK, in which exogenous application of CK show increased level of SA-dependent gene expression during pathogen infection (Albrecht and Argueso, 2017). In rice, external application of SA analogue benzothiadiazole S-methylester (BTH) and kinetin rapidly increased expression of defence genes *OsPR1b* and *PBZ1* (see Appendix), while application of either hormone alone did not show a meaningful increase in defence gene expression, nor did co-treatment with SA and several hormones (Jiang et al., 2013; Albrecht and Argueso, 2017).

CK is expected to be a systemic signal shimmering nutrient availability based on positive interaction between CK levels and nitrogen status, and enhanced nitrogen availability caused in higher JA biosynthesis and also downstream inducible antiherbivore genes in *Nicotiana attenuate* (Dervinis et al., 2010). CK and GA puts antagonistic influence on different developmental processes in the plant like; shoot and root elongation, cell differentiation, shoot regeneration, and meristem activity. CK inhibits the synthesis of GA and induces its deactivation and GA inhibits CK responses (Weiss and Ori, 2007).

There is also antagonism between CK and Auxin in plant immune defence. Elevated level of Auxin enhances susceptibility of *Arabidopsis* infection by *P. syringae* pv. tomato DC3000 by repressing PR1. Whereas resistance and induction of PR1 was seen with elevated CK level (Naseem and Dandekar, 2012). Reduced CK caused to ABA-promoted stomatal closure, thereby decreasing carbon uptake and assimilation under challenging conditions, the up-regulation of CK oxidase can also decrease carbon metabolism (Egamberdieva et al., 2017). CK can operate not only on the level of guard cell and alleviate closing effect of ABA, but they can also partially inhibit ABA accumulation caused by drought stress (Pospisilova et al., 2005).

The impact of the SA-JA/ET core defence signaling in plant-pathogen exposure has long been established. However, our understanding of the influence plant growth-promoting hormones like CK; exerts on modulating the central defence pathways is still lagging. A focus on this area of plant defence will reveal key biological inferences concerning the trade-off between defence and growth in plants.

### Practical Application of Cytokinin Mediated Plant Protection: Combining Biofertilization by Microalgae With Biocontrol and Improvement of Abiotic Stress Tolerance

The use of macroalgae in fields as a bio-fertilizer is a well-known practice (Prasanna et al., 2013; Righini et al., 2018; Uthirapandi et al., 2018). Cyanobacteria (prokaryotic green-blue algae) and microalgae (eukaryotic microalgae) are photosynthetic organisms and evolved from algae. Both found in water bodies, desert crusts, or even in symbiosis with other animals and share almost equal properties. Several previous studies have shown that microalgae can be pathogen resistance inducers. Using cyanobacteria in composts have been described as promoting resistance towards pathogens in tomato, cotton and zucchini (Dukare et al., 2011; Prasanna et al., 2013; Babu et al., 2015; Roberti et al., 2015). Different extracts of microalgal compounds have been used in interaction with pathogens to induce resistance, and is some cases how the extracts hinder growth of different pathogens directly (Jaulneau et al., 2010; Galal et al., 2011; Chowdhury et al., 2015; Stadnik and Freitas, 2014).

Interestingly, many articles point out that microalgae have been shown to produce Phytohormones, including auxin, ABA, CK, ET, and GAs. The presence of CKs have been reported in the extracts of Ulva (Sekar et al., 1995), *Durvillaea potatorum* and *Ascophyllum nodosum* (Craft et al., 2007) which stimulates early seedling growth in the plants. Pathogen response was shown to be dependent on JA signaling by ulvans, heteropolysaccharides from green algae of genus Ulva (Jaulneau et al., 2010). Enhanced growth and biochemical parameters of *Ocimum sanctum* reported by foliear spray of liquid extracts containing CK from marine macro *algae Sargassum wightii, Turbinaria ornata* and *Caulerpa racemosaon* (Uthirapandi et al., 2018). And furthermore, the five cyanobacteria *Anabaena, Oscillatoria, Phormidium, Chroococcidiopsis* and *Synechosystis* have been shown to CKs production (Hussain et al., 2010) whereas the level of protection was found to be similar to that determined by the CK producing bacterium *P. fluorescens* G20-18 (Grosskinsky et al., 2016).

## Concluding Remarks and Outlook

**Figure 1:**
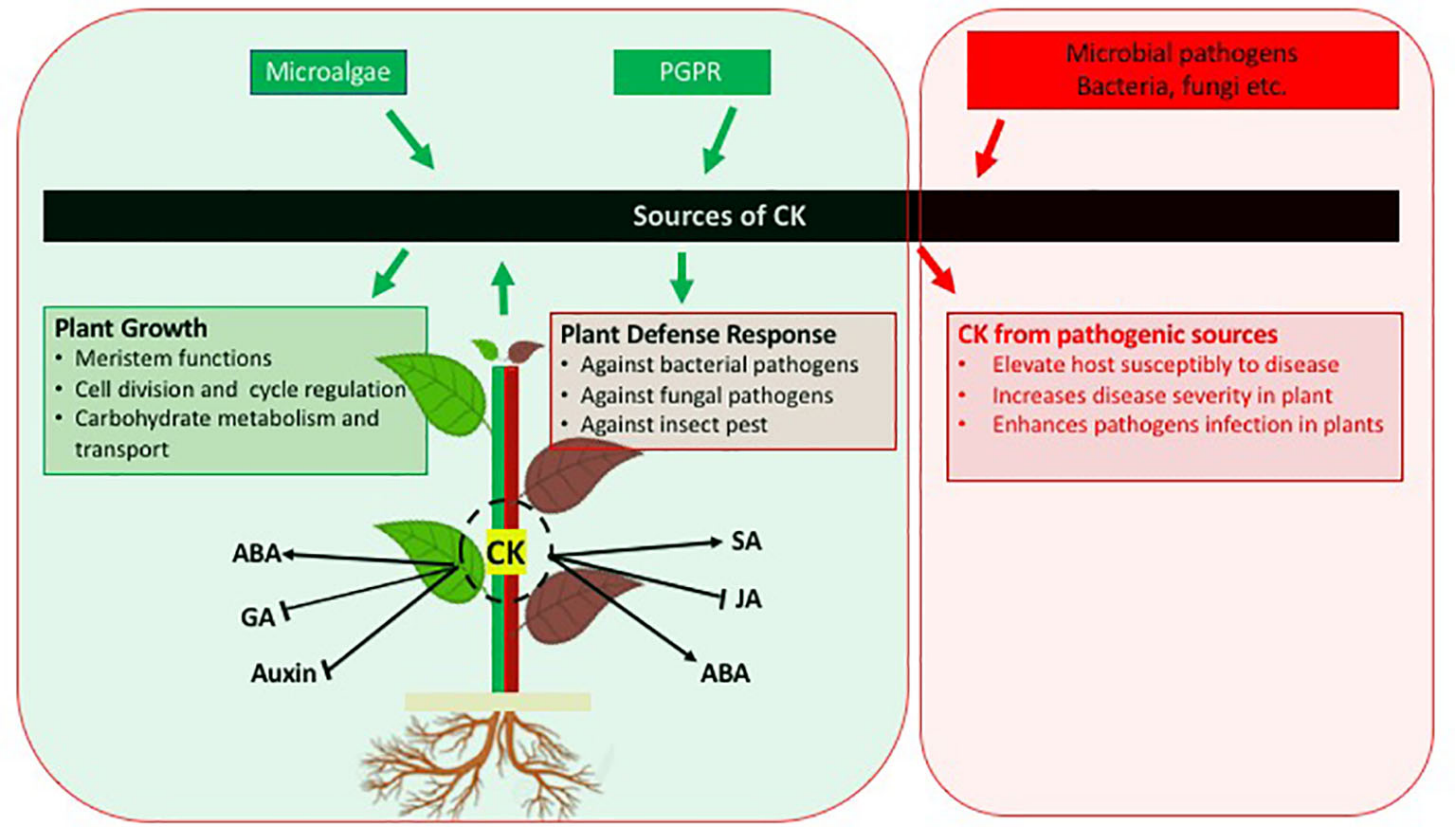
Schematic diagram indicating the role of cytokinins in plant growth and defence against pathogens. In addition, cross-talk between CK and other phytohormones is also presented. Arrows indicate positive interaction; blunt ends indicate negative interaction.

After a direct role of the classical plant growth stimulating phytohormone CK in the activation of plant defence had been recognized in the early 2010s, the production of CK by beneficial microorganisms is being recognized as relevant and widespread for inter-kingdom signaling to increase the immunity of plants against pathogens and pests (**Figure 1**). The microbial derived CKs are interacting with other plant hormone signaling pathways in a similar way as plant derived CKs thus being integrated in plant signaling networks and also inducing the direct synthesis of phytoalexins. Thus the CKs produced by bacteria and microalgae provide a protection against pathogens in a similar way as exogenously providing CKs or overexpressing the CK biosynthetic genes.

It is intriguing that beneficial microbes share different effects known to be stimulated by CKs, namely growth, abiotic stress tolerance and biocontrol. Thus it remains to be elucidated whether also other functions of beneficial microbes are actually mediated by microbial CK production. Thus the role of microbial CK production will have to be considered in the interaction of the root and shoot microbiome with plants and how the holobiont growth, physiology and abiotic and biotic stress resiliency is affected. Also at a mechanistic level there are still number of open questions. Since CKs are also contributing to resource allocation *via* stimulation of sink metabolism (Ehneß and Roitsch, 1997; Roitsch and Ehneß, 2000; Lara et al., 2004). The relative contributions of the indirect effect of physiological defence competence versus direct impact on defence mechanisms needs to be elucidated (Berger et al., 2007). Also the contradictory reports regarding the target species specificity of CK mediated biocontrol mechanisms will need to be clarified. Since CKs may be derived both from adenine or phenylurea and they comprise a variety of differentially modified molecules it needs to be clarified whether a structure—function specificity exists for eliciting the biocontrol effect (Grosskinsky et al., 2013). The latter aspect in particular applies to the fact that CKs are produced both by beneficial microbes as well as pathogens. It remains to be determined whether CKs produced by bacterial pathogens and their plant responses differ from those from beneficial microbes. To tackle the various open questions a holistic multi-omics approach (Großkinsky et al., 2017) will be need to be combined with dual functional approaches with targeted genetic modification in the pant and the microbe.

Virtually nothing is known how the production of CKs in microorganisms is regulated in response to environmental stimuli or plant derived signals. Thus it remains to be elucidated whether the plant derived factors differentially affect CK production in beneficial versus pathogenic microorganisms and whether the interference with CK production in pathogens is part of the plant defence.

The biocontrol ability of microbial derived CKs opens up promising applications in integrated crop plant protection based on biologicals. Notably the combination with other beneficial CKs effects, such as the improvement of abiotic stress tolerance, or of the CK producing microbes, such as the biofertilizer function of microalgae, seems to be attractive in environmentally friendly and sustainable approaches to reduce the use of chemical pesticides within climate change scenarios. The possible promising applications of CK producing microorganisms in crop plant management will have to be also considered in screening programs for new beneficial microbes or the composition of synthetic communities. In contrast to CK originated from beneficial microorganism source, CK synthesized by pathogenic organism have the opposite effect on plant growth and defence. However, further research is warranted to investigate the type and structure of CK molecules produced by both beneficial and pathogenic organisms to understand their plant-beneficial and plant detrimental mode of action.

## Author Contributions

All authors contributed equally to the article. SA, MM, CP and TR reviewed the literature and jointly wrote the article. SA, MM and CP contributed to manuscript revision and editing. TR provided feedback and final proof reading. SA coordinated the submission.

## Conflict of Interest

The authors declare that the research was conducted in the absence of any commercial or financial relationships that could be construed as a potential conflict of interest.

## Abbreviations

ABA, Abscisic acid; BR, Brassinosteroid; BTH, benzothiadiazole S-methyl ester; CK, Cytokinin; ET, Ethylene; GB/GA, Gibberellins/Gibberellic acid; JA, Jasmonic acid; PGPB, Plant growth promoting bacteria; Pst, Pseudomonas syringae pv tabaci; SA, Salicylic acid; SL, Strigolactone; Tzs, 6-(E)-(4-hydroxy-3-methylbut-2-enylamino)purine (trans-zeatin); TMV, Tobacco mosaic virus; BTH, benzothiadiazole S-methyl ester; PPFMs, Pink-pigmented facultative methylotrophs.

## Appendix

ARR: Arabidopsis response regulator
CKX (Cytokinin oxidases/Dehydrogenases): CKXs catalyze the degradation of cytokinins
IPT (Isopentenyl transferase): cytokinin synthase that mediates a key regulatory step during cytokinin biosynthesis
AHK (Arabidopsis histidine kinase): histidine kinase cytokinin receptors that transduce cytokinin signaling
OsPR1b: Pathogenesis-related (PR) protein in Rice (*Oryza sativa* L.)
PBZ1: Probenazole-Induced Protein1
PR1: pathogenesis-related protein 1
MYB: Myb transcription factor

## References

[B1] Akagi A. Fukushima S. Okada K. Jiang C. J. Yoshida R. Nakayama A. (2014). WRKY45-dependent priming of diterpenoid phytoalexin biosynthesis in rice and the role of cytokinin in trig- gering the reaction. Plant Mol. Biol. 86, 171–183. 10.1007/s11103-014-0221-x 25033935 PMC4133022

[B2] Akhtar S. S. Andersen M. N. Naveed M. Zahir Z. A. Liu F. (2015). Interactive effect of biochar and plant growth-promoting bacterial endophytes on ameliorating salinity stress in maize. Funct. Plant Biol. 4, 770–781. 10.1071/Fp15054 32480720

[B3] Albrecht T. Argueso C. T. (2017). Should I fight or should I grow now? the role of cytokinins in plant growth and immunity and in the growth-defence trade-off. Ann. Bot. 119, 725–735. 10.1093/aob/mcw211 27864225 PMC5379597

[B4] Angra-Sharma R. Sharma D. K. (2000). Cytokinins in pathogenesis and disease resistance of *Pyrenophora teres*-barley and *Dreschslera maydis*-maize interactions during early stages of infection. Mycopathologia 148, 87–95. 10.1023/a:1007126025955 11189749

[B5] Argueso C. T. Ferreira F. J. Epple P. To J. P. Hutchison C. E. Schaller G. E. (2012). Two-component elements mediate interactions between cytokinin and salicylic acid in plant immunity. PLoS Genet. 8, e1002448. 10.1371/journal.pgen.1002448 22291601 PMC3266875

[B6] Arkhipova T. N. Veselov S. U. Melentiev A. I. Martynenko E. V. Kudoyarova G. R. (2005). Ability of bacterium *Bacillus subtilis* to produce cytokinins and to influence the growth and endogenous hormone content of lettuce plants. Plant Soil 272, 201–209. 10.1007/s11104-004-5047-x

[B7] Arkhipova T. N. Prinsen E. Veselov S. U. Martinenko E. V. Melentiev A. I. Kudoyarova G. R. (2007). Cytokinin producing bacteria enhance plant growth in drying soil. Plant Soil 292, 305–315. 10.1007/s11104-007-9233-5

[B8] Babosha A. V. (2009). Regulation of resistance and susceptibility in wheat- powdery mildew pathosystem with exogenous cytokinins. J. Plant Physiol. 166, 1892–1903. 10.1371/journal.pgen.1002448 19592133

[B9] Babu S. Bidyarani N. Chopra P. Monga D. Kumar R. Prasanna R. (2015). Evaluating microbe-plant interactions and varietal differences for enhancing biocontrol efficacy in root rot disease challenged cotton crop. Eur. J. Plant Pathol. 142, 345–362. 10.1007/s10658-015-0619-6

[B10] Belimov A. A. Dodd I. C. Safronova V. I. Shaposhnikov A. I. Azarova T. S. Makarova N. M. (2015). Rhizobacteria that produce auxins and contain 1-amino-cyclopropane-1-carboxylic acid deaminase decrease amino acid concentrations in the rhizosphere and improve growth and yield of well-watered and water-limited potato (*Solanum tuberosum*). Ann. App. Biol. 167, 11–25. 10.1111/aab.12203

[B11] Bennett E. Roberts J. A. Wagstaff C. (2012). Manipulating resource allocation in plants. J. Exp. Bot. 63, 3391–3400. 10.1093/jxb/err442 22291133

[B12] Berens M. L. Berry H. M. Mine A. Argueso C. T. Tsuda K. (2017). Evolution of hormone signaling networks in plant defense. Ann. Rev. Phytopathol. 55, 401–425. 10.1146/annurev-phyto-080516-035544 28645231

[B13] Berger S. Sinha A. K. Roitsch T. (2007). Plant physiology meets phytopathology: plant primary metabolism and plant-pathogen interactions. J. Expt. Bot. 58, 4019–4026. 10.1093/jxb/erm298 18182420

[B14] Brütting C. Crava C. M. Schäfer M. Schuman M. C. Meldau S. Adam N. (2018). Cytokinin transfer by a free-living mirid to Nicotiana attenuata recapitulates a strategy of endophytic insects. Elife 7, e36268. 10.7554/eLife.36268 30014847 PMC6059766

[B15] Castillo P. Molina R. Andrade A. Vigliocco A. Alemano S. Cassán F. D. (2015). “Phytohormones and other plant growth regulators produced by PGPR: The genus *Azospirillum* ,” in Handbook for Azospirillum. Eds. Cassán F. Okon Y. Creus C. (Cham: Springer). 10.1007/978-3-319-06542-7_7

[B16] Chanclud E. Kisiala A. Emery N. R. Chalvon V. Ducasse A. Romiti-Michel C. (2016). Cytokinin production by the rice blast fungus is a pivotal requirement for full virulence. PLoS Pathog. 12, e1005457. 10.1371/journal.ppat.1005457 26900703 PMC4765853

[B17] Choi J. Hwang I. (2007). Cytokinin: perception, signal transduction, and role in plant growth and development. J. Plant Biol. 50, 98–108. 10.1007/BF03030617

[B18] Choi J. Huh S. U. Kojima M. Sakakibara H. Paek K. H. Hwang I. (2010). The Cytokinin-activated transcription factor ARR2 promotes plant immunity *via* TGA3/NPR1-dependent salicylic acid signaling in arabidopsis. Dev. Cell 19, 284–295. 10.1016/j.devcel.2010.07.011 20708590

[B19] Chowdhury M. M. H. Kubra K. Hossain M. B. Mustafa M. G. Jainab T. Karim M. R. (2015). Screening of antibacterial and antifungal activity of freshwater and marine algae as a prominent natural antibiotic available in Bangladesh. Int. J. Pharmacol. 11, 828–833. 10.3923/ijp.2015.828.833

[B20] Cordero I. Balaguer L. Rincon A. Pueyo J. J. (2018). Inoculation of tomato plants with selected PGPR represents a feasible alternative to chemical fertilization under salt stress. J. Plant Nut. Soil Sci. 181, 694–703. 10.1002/jpln.201700480

[B21] Cortleven A. Leuendorf J. E. Frank M. Pezzetta D. Bolt S. Schmulling T. (2019). Cytokinin action in response to abiotic and biotic stresses in plants. Plant Cell Environ. 42, 998–1018. 10.1111/pce.13494 30488464

[B22] Craft C. A. Hiltz D. A. Hankins S. D. MacKinnon S. L. (2007). Detection of plant growth hormones in Ascophyllum nodosum and seaweed products, in: MANAPRO XII, Proceedings of the 12th International Symposium on Marine Natural Products in Queenstown New Zealand, Oral-Poster Abstract, PO74-OR, 2007.

[B23] Davies P. J. (2010). “The plant hormones: their nature, occurrence, and functions,” in Plant hormones. Ed. Davies P. J. (Dordrecht: Springer), 1–15.

[B24] Dermastia M. (2019). Plant hormones in phytoplasma infected plants. Front. Plant Sci. 10, 477. 10.3389/fpls.2019.00477 31057582 PMC6478762

[B25] Dervinis C. Frost C. J. Lawrence S. D. Novak N. G. Davis J. M. (2010). Cytokinin primes plant responses to wounding and reduces insect performance. J. Plant Growth Regul. 29, 289–296. 10.1007/s00344-009-9135-2

[B26] Dowd C. D. Chronis D. Radakovic Z. S. Siddique S. Schmülling T. Werner T. (2017). Divergent expression of cytokinin biosynthesis, signaling and catabolism genes underlying differences in feeding sites induced by cyst and root-knot nematodes. Plant J. 92, 211–228. 10.1111/tpj.13647 28746737

[B27] Dukare A. S. Prasanna R. Dubey S. C. Chaudhary V. Nain L. Singh R. (2011). Evaluating novel microbe amended composts as biocontrol agents in tomato. Crop Prot. 30, 436–442. 10.1016/j.cropro.2010.12.017

[B28] Egamberdieva D. Wirth S. J. Alqarawi A. A. Abd_Allah E. F. Hashem A. (2017). Phytohormones and beneficial microbes: essential components for plants to balance stress and fitness. Front. Microbiol. 8, 1–14. 10.3389/fmicb.2017.02104 29163398 PMC5671593

[B29] Ehneß R. Roitsch T. (1997). Coordinated induction of extracellular invertase and glucose transporters in Chenopodium rubrum by cytokinins. Plant J. 11, 539–548. 10.1046/j.1365-313x.1997.11030539.x 9107040

[B30] Elzen G. W. (1983). Minireview: cytokinins and insect galls. Comp. Bioch. Physiol. 76, 17–19. 10.1016/0300-9629(83)90286-4

[B31] Engelbrecht L. Orban U. Heese W. (1969). Leaf-miner caterpillars and cytokinins in the “green islands” of autumn leaves. Nature 223, 319. 10.1038/223319a0

[B32] Galal H. R. M. Salem W. M. Nasr El-Deen F. (2011). Biological control of some pathogenic fungi using marine algae. Res. J. Microbiol. 6, 645–657. 10.3923/jm.2011.645.657

[B33] Giron D. Glevarec G. (2014). Cytokinin-induced phenotypes in plant-insect interactions: learning from the bacterial. World J. Chem. Ecol. 40, 826–835. 10.1007/s10886-014-0466-5 24944001

[B34] Giron D. Kaiser W. Imbault N. Casas J. (2007). Cytokinin-mediated leaf manipulation by a leafminer caterpillar. Biol. Lett. 3, 340–343. 10.1098/rsbl.2007.0051 17412674 PMC2390681

[B35] Giron D. Frago E. Glevarec G. Pieterse C. M. Dicke M. (2013). Cytokinins as key regulators in plant–microbe–insect interactions: connecting plant growth and defence. Funct. Ecol. 27, 599–609. 10.1111/1365-2435.12042

[B36] Giron D. Huguet E. Stone G. N. Body M. (2016). Insect-induced effects on plants and possible effectors used by galling and leaf-mining insects to manipulate their host-plant. J. Insect Physiol. 84, 70–89. 10.1016/j.jinsphys.2015.12.009 26723843

[B37] Glick B. R. Bashan Y. (1997). Genetic manipulation of plant growth-promoting bacteria to enhance biocontrol of phytopathogens. Biotech. Adv. 15, 353–378. 10.1016/S0734-9750(97)00004-9 14538716

[B38] Großkinsky D. K. Syaifullah S. J. Roitsch T. (2017). Integration of multi-omics techniques and physiological phenotyping within a holistic phenomics approach to study senescence in model and crop plants. J. Exp. Bot. 66, 825–844. 10.1093/jxb/erx333 29444308

[B39] Grosskinsky D. K. Naseem M. Abdelmohsen U. R. Plickert N. Engelke T. Griebel T. (2011). Cytokinins mediate resistance against *Pseudomonas syringae* in tobacco through increased antimicrobial phytoalexin synthesis independent of salicylic acid signaling. Plant Physiol. 157, 815–830. 10.1104/pp.111.182931 21813654 PMC3192561

[B40] Grosskinsky D. B. Edelsbrunner K. Pfeifhofer H. v. d. Graaff E. Roitsch T. (2013). Cis- and trans-zeatin differentially modulate plant immunity. Plant Signal. Behav. 8:7, e24798. 10.4161/psb.24798 23656869 PMC3906432

[B41] Grosskinsky D. K. Tafner R. Moreno M. V. Stenglein S. A. de Salamone I. E. G. Nelson L. M. (2016). Cytokinin production by Pseudomonas fluorescens G20-18 determines biocontrol activity against Pseudomonas syringae in Arabidopsis. Sci. Rep. 6, 23310. 10.1038/srep23310 26984671 PMC4794740

[B42] Guo Y. Gan S. (2014). Translational researches of leaf senescence for enhancing plant productivity and quality. J. Exp. Bot. 65, 3901–3913. 10.1093/jxb/eru248 24935620

[B43] Gururani M. A. Upadhyaya C. P. Baskar V. Venkatesh J. Nookaraju A. Park S. W. (2013). Plant growth-promoting rhizobacteria enhance abiotic atress tolerance in *Solanum tuberosum* through inducing changes in the expression of ROS-Scavenging enzymes and improved photosynthetic performance. J. Plant Growth Regul. 32, 245–258. 10.1007/s00344-012-9292-6

[B44] Hinsch J. Vrabka J. Oeser B. Novák O. Galuszka P. Tudzynski P. (2015). De novo biosynthesis of cytokinins in the biotrophic fungus *Claviceps purpurea* . Environ. Microbiol. 17, 2935–2951. 10.1111/1462-2920.12838 25753486

[B45] Holland M. A. (1997). Occam's razor applied to hormonology. Plant Physiol. 115, 865–868. 10.1104/pp.115.3.865 12223849 PMC158548

[B46] Hui D. Iqbal J. Lehmann K. Gase K. Saluz H. P. Baldwin I. T. (2003). Molecular Interactions between the specialist herbivoremanduca sexta (*Lepidoptera, Sphingidae*) and its natural host nicotiana attenuata: V. Microarray analysis and further characterization of large-scale changes in herbivore-induced mRNAs. Plant Physiol. 131, 1877–1893. 10.1104/pp.102.018184 12692347 PMC166944

[B47] Hussain A. Krischke M. Roitsch T. Hasnain S. (2010). Rapid determination of cytokinins and auxin in cyanobacteria. Curr. Microbiol. 6, 361–369. 10.1007/s00284-010-9620-7 20339849

[B48] Hwang H. H. Wang M. H. Lee Y. L. Tsai Y. L. Li Y. H. Yang F. J. (2010). Agrobacterium-produced and exogenous cytokinin-modulated Agrobacterium-mediated plant transformation. Mol. Plant Pathol. 11, 677–690. 10.1111/j.1364-3703.2010.00637.x 20696005 PMC6640272

[B49] Hwang I. Sheen J. Müller B. (2012). Cytokinin signaling networks. Annu. Rev. Plant Biol. 63, 353–380. 10.1146/annurev-arplant-042811-105503 22554243

[B50] Ishizawa H. Kuroda M. Inoue K. Inoue D. Morikawa M. Ike M. (2019). Colonization and competition dynamics of plant growth-promoting/inhibiting bacteria in the phytosphere of the duckweed lemna minor. Microb. Ecol. 77, 440–450. 10.1007/s00248-018-1306-x 30603770

[B51] Jameson P. E. Dhandapani P. Song J. Zatloukal M. Strnad M. Remus-Emsermann M. N. (2019). The cytokinin complex associated with *Rhodococcus fascians*: which compounds are critical for virulence? Front. Plant Sci. 10, 674. 10.3389/fpls.2019.00674 31191583 PMC6539147

[B52] Jameson P. E. (2000). Cytokinins and Auxins in plant-pathogen interactions - an overview. Plant Growth Regul. 32, 369–380. 10.1023/A:1010733617543

[B53] Jaulneau V. Lafitte C. Jacquet C. Fournier S. Salamagne S. Briand X. (2010). Ulvan, a sulfated polysaccharide from green algae, activates plant immunity through the jasmonic acid signaling pathway. J. Biomed. Biotechnol. 2010, 525291. 10.1155/2010/525291 20445752 PMC2860583

[B54] Jiang C.-J. Shimono M. Sugano S. Kojima M. Liu X. Inoue H. (2013). Cytokinins act synergistically with salicylic acid to activate defense gene expression in rice. Mol. Plant. Microbe Interact. 26, 287–296. 10.1094/MPMI-06-12-0152-R 23234404

[B55] Jorge G. L. Kisiala A. Morrison E. Aoki M. Nogueira A. P. O. Emery R. J. N. (2019). Endosymbiotic Methylobacterium oryzae mitigates the impact of limited water availability in lentil (Lens culinaris Medik.) by increasing plant cytokinin levels. Env. Exp. Bot. 162, 525–540. 10.1016/j.envexpbot.2019.03.028

[B56] Joseph J. Kieber G. Schaller E. (2018). Cytokinin signaling in plant development. Development 145, dev149344. 10.1242/dev.149344 29487105

[B57] Kaiser W. Huguet E. Casas J. Commin C. Giron D. (2010). Plant green-island phenotype induced by leaf-miners is mediated by bacterial symbionts. Proc. Royal Soc. B. Biol. Sci. 277, 2311–2319. 10.1098/rspb.2010.0214 PMC2894905 20356892

[B58] Kaminek M. (2015). Tracking the story of cytokinin research. J. Plant Growth Regul. 34, 723–739. 10.1007/s00344-015-9543-4

[B59] Kanwal S. Ilyas N. Batool N. Arshad M. (2017). Amelioration of drought stress in wheat by combined application of PGPR, compost, and mineral fertilizer. J. Plant Nutr. 40, 1250–1260. 10.1080/01904167.2016.1263322

[B60] Kind S. Hinsch J. Vrabka J. Hradilová M. Majeská-Čudejková M. Tudzynski P. Galuszka P (2018). Manipulation of cytokinin level in the ergot fungus Claviceps purpurea emphasizes its contribution to virulence. Curr. Gent. 64, 1303–1319. 10.1007/s00294-018-0847-3 29850931

[B61] Kumar M. Kour D. Yadav A. N. Saxena R. Rai P. K. Jyoti A. (2019). Biodiversity of methylotrophic microbial communities and their potential role in mitigation of abiotic stresses in plants. Biologia 74, 287–308. 10.2478/s11756-019-00190-6

[B62] Lara M. E. B. Garcia M. C. G. Fatima T. Ehneß R. Lee T. K. Proels R. (2004). Extracellular invertase is an essential component of cytokinin-mediated delay of senescence. Plant Cell 16, 1276–1287. 10.1105/tpc.018929 15100396 PMC423215

[B63] Lisabeth E. (1971). Cytokinin activity in larval infected leaves. Biochem. Physiol. Pflanz. 162, 9–27. 10.1016/S0015-3796(17)31102-2

[B64] Liu F. C. Xing S. J. Ma H. L. Du Z. Y. Ma B. Y. (2013). Cytokinin-producing, plant growth-promoting rhizobacteria that confer resistance to drought stress in Platycladus orientalis container seedlings. App. Microb. Biotech. 97, 9155–9164. 10.1007/s00253-013-5193-2 23982328

[B65] Liu Z. Bushnell W. R. (1986). Effects of cytokinins on fungus development and host response in powdery mildew of barley. Physiol. Mol. Plant Pathol. 29 (1), 47–52. 10.1016/S0048-4059(86)80036-4

[B66] Maheshwari D. K. Dheeman S. Agarwal M. (2015). “Phytohormone-producing PGPR for sustainable sgriculture,” in Bacterial Metabolites in Sustainable Agroecosystem. Sustainable Development and Biodiversity, vol. 12 Ed. Maheshwari D. (Cham: Springer), 159–182. 10.1007/978-3-319-24654-3_7

[B67] Maksimov I. V. Abizgil'dina R. R. Pusenkova L. I. (2011). Plant growth promoting rhizobacteria as alternative to chemical crop protectors from pathogens (review). Appl. Biochem. Micro. 47, 333–345. 10.1134/S0003683811040090 21950110

[B68] Mapes C. C. Davies P. J. (2001). Cytokinins in the ball gall of Solidago altissima and in the gall forming larvae of Eurosta solidaginis. New Phytol. 151, 203–212. 10.1046/j.1469-8137.2001.00158.x 33873383

[B69] Mishra V. Ellouze W. Howard R. J. (2018). Utility of *Arbuscular Mycorrhizal* fungi for improved production and disease mitigation in organic and hydroponic greenhouse crops. J. Hortic. 5, 2376–0354. 10.4172/2376-0354.1000237

[B70] Moreno J. E. Ballaré C. L. (2014). Phytochrome regulation of plant immunity in vegetation canopies. J. Chem. Ecol. 40, 848–857. 10.1007/s10886-014-0471-8 25063023

[B71] Morrison E. N. Emery R. N. Saville B. J. (2015). Phytohormone involvement in the Ustilago maydis–Zea mays pathosystem: relationships between abscisic acid and cytokinin levels and strain virulence in infected cob tissue. PloS One 10, e0130945. 10.1371/journal.pone.0130945 26107181 PMC4479884

[B72] Munné-Bosch S. Müller M. (2013). Hormonal cross-talk in plant development and stress responses. Front. Plant Sci. 4, 529. 10.3389/fpls.2013.00529 24400014 PMC3870953

[B73] Nadeem S. M. Ahmad M. Zahir Z. A. Javaid A. Ashraf M. (2014). The role of mycorrhizae and plant growth promoting rhizobacteria (PGPR) in improving crop productivity under stressful environments. Biotechnol. Adv. 32, 429–448. 10.1016/j.biotechadv.2013.12.005 24380797

[B74] Nadeem S. M. Imran M. Naveed M. Khan M. Y. Ahmad M. Zahir Z. A. (2017). Synergistic use of biochar, compost and plant growth-promoting rhizobacteria for enhancing cucumber growth under water deficit conditions. J. Sci. Food Agric. 97, 5139–5145. 10.1002/jsfa.8393 28436040

[B75] Naseem M. Dandekar T. (2012). The role of auxin-cytokinin antagonism in plant-pathogen interactions. PLoS Pathog. 8, e1003026. 10.1371/journal.ppat.1003026 23209407 PMC3510258

[B76] Naseem M. Kaltdorf M. Hussain A. Dandekar T. (2013). The impact of cytokinin on jasmonate-salicylate antagonism in Arabidopsis immunity against infection with Pst DC3000. Plant Signal. Behav. 8, e26791. 10.4161/psb.26791 PMC4091086 24494231

[B77] Naseem M. Wolfling M. Dandekar T. (2014). Cytokinins for immunity beyond growth, galls and green islands. Trends Plant Sci. 19, 481–484. 10.1016/j.tplants.2014.04.001 24794463

[B78] Novák J. Pavlů J. Novák O. Nožková-Hlaváčková V. Špundová M. Hlavinka J. (2013). High cytokinin levels induce a hypersensitive-like response in tobacco. Ann. Bot. 112, 41–55. 10.1093/aob/mct092 23644362 PMC3690983

[B79] O'Brien J. A. Benková E. (2013). Cytokinin cross-talking during biotic and abiotic stress responses. Front. Plant Sci. 4, 451. 10.3389/fpls.2013.00451 24312105 PMC3833016

[B80] Pangesti N. Pineda A. Pieterse C. M. Dicke M. Van-Loon J. J. (2013). Two-way plant mediated interactions between root-associated microbes and insects: from ecology to mechanisms. Front. Plant Sci. 4, 1–11. 10.3389/fpls.2013.00414 24167508 PMC3805956

[B81] Pieterse C. M. Zamioudis C. Berendsen R. L. Weller D. M. Van Wees S. C. Bakker P. A. (2014). Induced systemic resistance by beneficial microbes. Ann. Rev. Phytopathol. 52, 347–375. 10.1146/annurev-phyto-082712-102340 24906124

[B82] Pospisilova J. Vagner M. Malbeck J. Travnickova A. Batkova P. (2005). Interactions between abscisic acid and cytokinins during water stress and subsequent rehydration. Biol. Plant. 49, 533–540. 10.1007/s10535-005-0047-0

[B83] Prasanna R. Chaudhary V. Gupta V. Babu S. Kumar A. Singh R. (2013). Cyanobacteria mediated plant growth promotion and bioprotection against Fusarium wilt in tomato. Eu. J. Plant Pathol. 136, 337–353. 10.1007/s10658-013-0167-xs

[B84] Pusztahelyi T. Holb I. J. Pócsi I. (2016). “Plant-Fungal Interactions: Special secondary metabolites of the biotrophic, necrotrophic, and other specific interactions,” in Fungal metabolites. Reference Series in Phytochemistry. Eds. Mérillon J. M. Ramawat K. (Cham: Springer), 133–190. 10.1007/978-3-319-19456-1_39-1

[B85] Regier D. A. Morris R. O. (1982). Secretion of trans-zeatin by agrobacterium-Tumefaciens - a function determined by the nopaline ti plasmid. Biochem. Biophys. Res. Commun. 104, 1560–1566. 10.1016/0006-291x(82)91429-2 7073755

[B86] Righini H. Roberti R. Baraldi E. (2018). Use of algae in strawberry management. J. Appl. Phycol. 30, 3551–3564. 10.1007/s10811-018-1478-2

[B87] Roitsch T. Ehneß R. (2000). Regulation of source/sink relations by cytokinins. Plant Growth Regul. 32, 359–267. 10.1023/A:1010781500705

[B88] Romanov G. A. (2011). The discovery of cytokinin receptors and biosynthesis of cytokinins: a true story. R. J. Plant Physiol. 58, 743–747. 10.1134/S1021443711040121.pdf

[B89] Ryu C. M. Farag M. A. Hu C. H. Reddy M. S. Kloepper J. W. Pare P. W. (2004). Bacterial volatiles induce systemic resistance in *Arabidopsis* . Plant Physiol. 134, 1017–1026. 10.1104/pp.103.026583 14976231 PMC389924

[B90] Sørensen J. L. Benfield A. H. Wollenberg R. D. Westphal K. Wimmer R. Nielsen M. R. (2018). The cereal pathogen Fusarium pseudograminearum produces a new class of active cytokinins during infection. Mol. Plant Pathol. 19, 1140–1154. 10.1111/mpp.12593 28802024 PMC6638160

[B91] Sakakibara H. (2006). Cytokinins: activity, biosynthesis, and translocation. Annu. Rev. Plant Biol. 57, 431–449. 10.1146/annurev.arplant.57.032905.105231 16669769

[B92] Sardesai N. Lee L. Y. Chen H. B. Yi H. C. Olbricht G. R. Stirnberg A. (2013). Cytokinins Secreted by agrobacterium promote transformation by repressing a plant Myb transcription factor. Sci. Signal. 6, ra100. 10.1126/scisignal.2004518 24255177

[B93] Schäfer M. Meza-Canales I. D. Navarro-Quezada A. Brütting C. Vanková R. Baldwin I. T. (2015). Cytokinin levels and signaling respond to wounding and the perception of herbivore elicitors in Nicotiana attenuata. J. Integ. Plant Biol. 57, 198–212. 10.1111/jipb.12227 PMC4286249 24924599

[B94] Sekar R. Thangaraju N. Rengasamy R. (1995). Effect of seaweed liquid fertilizer from *Ulva lactuca L.* on *Vigna unguiculata L.* (Walp). Phykos 34, 49–53. 10.1007/s10811-017-1082-x

[B95] Shanks C. M. Rice J. H. Yan Z. B. Schaller G. E. Hewezi T. Kieber J. J. (2016). The role of cytokinin during infection of Arabidopsis thaliana by the cyst nematode Heterodera schachtii. Mol. Plant-Microbe Interact. 29, 57–68. 10.1094/MPMI-07-15-0156-R 26479273

[B96] Siddique S. Radakovic Z. S. Carola M. Chronis D. Novák O. Ramireddy E. (2015). A parasitic nematode releases cytokinin that controls cell division and orchestrates feeding site formation in host plants. Proc. Nat. Acad. Sci. 112, 12669–12674. 10.1073/pnas.1503657112 26417108 PMC4611629

[B97] Song J. Jiang L. Jameson P. E. (2015). Expression patterns of Brassica napus genes implicate IPT, CKX, sucrose transporter, cell wall invertase, and amino acid permease gene family members in leaf, flower, silique, and seed development. J. Exp. Botany. 66, 5067–5082. 10.1093/jxb/erv133 25873685 PMC4513924

[B98] Spallek T. Melnyk C. W. Wakatake T. Zhang J. Sakamoto Y. Kiba T. (2017). Interspecies hormonal control of host root morphology by parasitic plants. Proc. Nat. Acad. Sci. 114, 5283–5288. 10.1073/pnas.1619078114 28461500 PMC5441792

[B99] Spallek T. Gan P. Kadota Y. Shirasu K. (2018). Same tune, different song—cytokinins as virulence factors in plant–pathogen interactions? Curr. Opin. Plant Biol. 44, 82–87. 10.1016/j.pbi.2018.03.002 29555490

[B100] Stadnik M. J. Freitas M. B. D. (2014). Algal polysaccharides as source of plant resistance inducers. Trop. Plant Pathol. 39, 111–118. 10.1590/S1982-56762014000200001

[B101] Su Y. Xia S. Wang R. Xiao L. (2017). “Phytohormonal quantification based on biological principles,” in Hormone Metabolism and Signaling in Plants. Eds. Li J. Li C. Smith S. M. (London, UK: Academic Press), 431–470.

[B102] Uthirapandi V. Suriya S. Boomibalagan P. Eswaran S. Ramya S. S. Vijayanand N. (2018). Bio-fertilizer potential of seaweed liquid extracts of marine macro algae on growth and biochemical parameters of Ocimum sanctum. J. Pharmacogn. Phytochem. 7, 3528–3532. archives/2018/vol7issue3/PartAV/7-3-244-742.pdf

[B103] Vrabka J. Niehaus E. M. Münsterkötter M. Proctor R. H. Brown D. W. Novák O. (2018). Production and role of hormones during interaction of Fusarium species with maize (*Zea mays* L.) seedlings. Front. Plant Sci. 9, 1936. 10.3389/fpls.2018.01936 30687345 PMC6337686

[B104] Walters D. R. McRoberts N. Fitt B. D. (2008). Are green islands red herrings? Significance of green islands in plant interactions with pathogens and pests. Biol. Rev. 83, 79–102. 10.1111/j.1469-185X.2007.00033.x 18093233

[B105] Wang C. J. Yang W. Wang C. Gu C. Niu D. D. Liu H. X. (2012). Induction of drought tolerance in cucumber plants by a consortium of three plant growth-promoting rhizobacterium strains. PLoS ONE 7, e52565. 10.1371/journal.pone.0052565 23285089 PMC3532358

[B106] Weiss D. Ori N. (2007). Mechanisms of cross talk between gibberellin and other hormones. Plant Physiol. 144, 1240–1246. 10.1104/pp.107.100370 17616507 PMC1914132

[B107] Zhang H. Guiguet A. Dubreuil G. Kisiala A. Andreas P. Emery R. N. (2017). Dynamics and origin of cytokinins involved in plant manipulation by a leaf-mining insect. Insect Sci. 24, 1065–1078. 10.1111/1744-7917.12500 28636152

[B108] Zhang H. Dubreuil G. Faivre N. Dobrev P. Kaiser W. Huguet E. (2018). Modulation of plant cytokinin levels in the *Wolbachia*-free leaf-mining species. Phyllonorycter Mespilella. Entomolog. Exp. Appl. 16, 428–438. 10.1111/eea.12681

[B109] Zhou C. Zhu L. Xie Y. Li F. Y. Xiao X. Ma Z. Y. (2017). Bacillus licheniformis SA03 confers increased saline-alkaline tolerance in chrysanthemum plants by induction of abscisic acid accumulation. Front. Plant Sci. 8, 1143. 10.3389/fpls.2017.01143 28706529 PMC5489591

